# Associations of Dietary Inflammatory Index Scores with the disability status and subjective health of older adults living in non-urban municipalities in Nagasaki and Ishikawa Prefectures, Japan

**DOI:** 10.1017/S1368980025000424

**Published:** 2025-03-31

**Authors:** Momoka Masuda, Chiho Goto, Hideki Imai, Shihomi Sakurai, Mikie Hidaka, Haruna Ushimura, Rieko Nakao, Mayumi Ohnishi, Masahiro Umezaki

**Affiliations:** 1 Department of Human Ecology, School of International Health, Graduate School of Medicine, The University of Tokyo, 7-3-1 Hongo, Bunkyo-ku, Tokyo 113-0033, Japan; 2 Department of Health and Nutrition, School of Health and Human Life, Nagoya Bunri University, 365 Maeda, Inazawa-cho, Inazawa-shi, Aichi 492-8520, Japan; 3 Faculty of Nursing, Ishikawa Prefectural Nursing University, 1-1 Gakuendai, Kahoku-shi, Ishikawa 929-1210, Japan; 4 School of Nursing, Tokyo Medical University, 6-1-1 Shinjuku, Shinjuku-ku, Tokyo 160-8402, Japan; 5 Nagasaki University Graduate School of Biomedical Sciences, 1-7-1 Sakamoto, Nagasaki-shi, Nagasaki 852-8520, Japan

**Keywords:** Dietary Inflammatory Index, Disability, Subjective health, Older people, Japan

## Abstract

**Objective:**

To examine associations of Dietary Inflammatory Index (DII) scores with disability and subjective health, which is prognostic of disability, in a large, systematically sampled population of older adults living in non-urban areas in Japan.

**Design:**

Cross-sectional. The Tokyo Metropolitan Institute of Gerontology Index of Competence was used to assess disability. Both overall disability and disabilities in components of everyday competence (instrumental activities of daily living (IADL), intellectual activities and social participation) were examined. Participants who reported an inability to perform one or more activities were categorised as disabled. Subjective health was assessed based on the response to the following question: ‘In general, how do you feel about your own health?’

**Setting:**

Six non-urban municipalities in Japan that differ in terms of regional characteristics.

**Participants:**

Adults aged 65–74 years (*n* 7930).

**Results:**

DII scores were positively associated with the odds of overall disability (OR (95 % CI)) = 1·23 (1·19, 1·28)); disabilities in IADL (OR (95 % CI) = 1·10 (1·05, 1·15)); intellectual activities (OR (95 % CI) = 1·28 (1·23, 1·33)); social participation (OR (95 % CI) = 1·17 (1·13, 1·22)) and poor subjective health (OR (95 %CI) = 1·09 (1·05, 1·14)).

**Conclusions:**

Our results imply the importance of reducing dietary inflammation to prevent both disability and a decline in subjective health, a predictor of disability.

Chronic low-grade inflammation, also termed ‘inflammaging’, plays a crucial role in aging, triggering the development of various age-related diseases and functional decline^(1,2)^. Dietary components (e.g. nutrients or food items) either promote or suppress inflammation by influencing the immune system^(3–5)^. The balance between pro- and anti-inflammatory dietary components determines overall dietary inflammatory status, which can be quantified using the Dietary Inflammatory Index (DII)^(6)^. Research on the relationship between DII scores and disability, conducted both in Japan and elsewhere, has focussed principally on impairments in basic physical and cognitive functions^(7–13)^. Our previous study added to existing knowledge by revealing an association between DII scores and reduced everyday competence (the ability to engage in complex processes that are indispensable when living independently). In increasing order of complexity, everyday competence includes instrumental activities of daily living (IADL), intellectual activities and social participation^(14)^. This implies that dietary inflammation should be reduced to prevent declines not only in basic physical and cognitive functions but also in the complex practical activities that require such functions in the older population.

However, our earlier study had certain limitations. The sample size was relatively small (*n* 1642), and sampling was not systematic; the recruitment procedures differed among the five participating municipalities. Also, examination of whether DII scores are associated with subjective health would reveal the extent to which dietary inflammation affects the predictors of disability development^(15,16)^. Subjective health is a subjective assessment of health status, encompassing both mental and physical well-being. A systematic review revealed that subjective health was one of the most important prognostic factors in IADL disability, which considers the most basic components of everyday competence^(16)^. A recent longitudinal study in Korea also suggested that poor subjective health predicted future IADL disability^(15)^.

Based on our previous study, we systematically sampled a larger number of older adults living in non-urban municipalities in Japan to examine the link between DII scores and disability in everyday competence. We enrolled a more general population of older Japanese adults from six municipalities that vary in terms of regional characteristics. We also included subjective health as an outcome, which has been suggested to predict IADL disability. As in our previous study, we also explored the various determinants of DII scores^(14)^.

## Methods

### Participants

Participants were recruited from six non-urban municipalities that differed in terms of regional characteristics. The municipalities were those of two Japanese prefectures: Suzu-shi in Ishikawa Prefecture and Unzen-shi, Shimabara-shi, Hasami-cho, Matsuura-shi and Minamishimabara-shi in Nagasaki Prefecture. The inclusion criteria were all residents of the target municipalities who were enrolled in the National Health Insurance system and aged 40–74 years at the time of the survey. For Shimabara-shi and Unzen-shi, of the residents who met the inclusion criteria, 50 % were randomly selected (with consideration of age and sex) and sent questionnaires due to the relatively larger population size and budgetary constraints. For the other municipalities, questionnaires were sent to all residents who met the inclusion criteria. Although this discrepancy in the sampling strategy could potentially affect the representativeness of the descriptive statistics of the entire study population, it should have minimal impact on the association analyses and the comparability between municipalities, as the eligible individuals in Shimabara-shi and Unzen-shi were randomly selected. Questionnaires were distributed in Hasami-cho, Matsuura-shi and Minamishimabara-shi in 2020, in Unzen-shi and Shimabara-shi in 2021 and in Suzu-shi in 2022. Questionnaires were sent to 30 558 eligible individuals across the six municipalities; 14 127 responded (46 %). After excluding individuals aged ≤ 64 years (*n* 4662), those aged ≥ 75 years (*n* 158), those with missing questionnaire data (*n* 1299) and those with energy intakes > 3 standard deviations from the mean (*n* 78), 7930 individuals were included in analysis.

### Dietary Inflammatory Index

DII scores were calculated based on the intakes of twenty nutrients^(14)^ detailed using a short FFQ validated for middle-aged and older Japanese adults^(17)^. DII scores were calculated via the following steps: using a global dietary database^(6)^, nutrient intakes were converted to Z-scores and then to percentile scores that were in turn multiplied by the ‘inflammatory effect scores’ of the nutrients to yield ‘food parameter-specific DII scores’, which were summed to obtain the final DII scores. Previous studies have validated the use of DII scores in Japanese populations^(18–20)^.

### Tokyo Metropolitan Institute of Gerontology Index of Competence

The Tokyo Metropolitan Institute of Gerontology Index of Competence is widely used to assess the everyday competence of Japanese populations^(21)^. The index covers three components of competence: IADL, intellectual activities and social participation. Disabilities in everyday competence reflect the early development of disability (as explained elsewhere^(14)^). The Tokyo Metropolitan Institute of Gerontology Index of Competence questionnaire explores the ability to perform thirteen activities in the abovementioned three categories. Overall disability and disabilities in each component of competence were assessed in this study. In terms of overall disability, a participant was considered ‘disabled’ if s/he could not perform at least one of the thirteen activities. Similarly, in terms of disability in each component, a participant was considered ‘disabled’ if s/he could not perform at least one of the activities included in the corresponding component. Those lacking any disability were considered ‘healthy’ in terms of both overall disability and disability in each component.

### Subjective health

Subjective health was assessed based on the answer to the following question: ‘In general, how do you feel about your own health?’ The responses were provided via a four-point scale: (1) excellent, (2) good, (3) poor and (4) very poor. Participants who reported excellent or good health were combined into the ‘good subjective health’ group, and all other participants comprised the ‘poor subjective health’ group. Poor subjective health, as assessed by a single-item question, has been associated with mortality in a meta-analysis of community-based prospective cohort studies from multiple countries published in English^(22)^, as well as in a longitudinal study among older adults in rural Japan^(23)^, providing evidence that supports the predictive validity of this single-item question. Three studies conducted in Australia^(24)^, Sweden^(25)^ and the USA^(26)^ have demonstrated moderate reliability within repeated assessments of subjective health using a single-item question.

### Statistical analysis

Factors influencing DII scores were sought using two regression models: Model 1 examined the association of each individual variable with the DII score, whereas Model 2 included all variables simultaneously. The variables included were sex (male, female); age (years); BMI (kg/m^2^); municipality (Suzu-shi, Unzen-shi, Shimabara-shi, Hasami-cho, Matsuura-shi and Minamishimabara-shi); living alone (yes, no); education level (≤ 9 years, ≥ 10 years); economic status (constrained, normal or good) and frequency of shopping (1–2 times per week or less, 3–6 times per week or more). Energy intake was included as a covariate in both models.

Subsequently, the associations between DII scores and everyday competence (*i.e.* overall disability and disability in each component of competence) and subjective health were examined via logistic regression analysis. The OR for being ‘disabled’ or having ‘poor subjective health’ were calculated. The covariates were sex, age, BMI, economic status, education level and energy intake. Analyses stratified by municipality were also conducted to examine the robustness of the results.

All statistical analyses were performed using R ver. 4·3·1 (R Project for Statistical Computing, Vienna, Austria). The significance level was set to *P* < 0·05.

## Results

Table 1 shows the descriptive statistics. The mean (sd) age was 69·9 (2·7) years, and the mean (sd) DII score was 0·19 (1·36). In total, 60 % of the participants exhibited overall disability, and 18 %, 37 % and 41 % had disabilities in IADL, intellectual activities and social participation, respectively; 23 % reported poor subjective health.

**Table 1:** Descriptive statistics of participants’ characteristics, Dietary Inflammatory Index (DII) scores^(6)^, disability assessed using the Tokyo Metropolitan Institute of Gerontology Index of Competence (TMIG-IC)^(21)^ and subjective health status (*n* 7930)

	Mean	sd
Age (years)	69·9	2·7
BMI (kg/m^2^)	23·0	4·5
DII score	0·19	1·36
	*n*	%
Sex		
Male	3630	46
Female	4300	54
TMIG-IC score (overall)		
Healthy	3183	40
Disabled	4747	60
TMIG-IC score (IADL)		
Healthy	6468	82
Disabled	1462	18
TMIG-IC score (intellectual activities)		
Healthy	4971	63
Disabled	2959	37
TMIG-IC score (social participation)		
Healthy	4660	59
Disabled	3270	41
Subjective health		
Good	6124	77
Poor	1806	23
Municipality		
Ishikawa prefecture		
Suzu-shi	1245	16
Nagasaki prefecture		
Unzen-shi	823	10
Shimabara-shi	1025	13
Hasami-cho	782	10
Matsuura-shi	1253	16
Minamishimabara-shi	2802	35
Living alone		
No	6879	87
Yes	1051	13
Education		
≤ 9 years	2195	28
≥ 10 years	5735	72
Economic status		
Constrained	2556	32
Normal or good	5374	68
Frequency of shopping		
1–2 times per week or less	3487	44
3–6 times per week or more	4443	56

IADL, instrumental activities of daily living.

The associations of participants’ characteristics with the DII scores are shown in Table 2. In the model that included all variables (Model 2), they were all associated with the DII scores. Female sex, older age, more education, higher economic status and more frequent shopping were associated with lower DII scores. A higher BMI, living alone and residency in Matsuura-shi or Minamishimabara-shi were associated with higher DII scores.

**Table 2:** Associations between participants’ characteristics and Dietary Inflammatory Index (DII) scores^(6)^ (*n* 7930)

	Model 1^*^		Model 2^†^	
	*β*	95 % CI	*β*	95 % CI
Sex				
Male (ref)				
Female	−0·75	−0·81, –0·68	−0·74	−0·80, –0·68
Age (years)	−0·05	−0·06, –0·04	−0·05	−0·06, –0·04
BMI (kg/m^2^)	0·02	0·01, 0·03	0·01	0·00, 0·02
Municipality				
Ishikawa Prefecture				
Suzu-shi (ref)				
Nagasaki prefecture				
Unzen-shi	0·07	−0·05, 0·19	0·08	−0·03, 0·20
Shimabara-shi	−0·01	−0·13, 0·10	0·05	−0·05, 0·16
Hasami-cho	−0·04	−0·16, 0·08	0·04	−0·08, 0·15
Matsuura-shi	0·15	0·04, 0·25	0·14	0·04, 0·24
Minamishimabara-shi	0·06	−0·03, 0·15	0·09	0·00, 0·17
Living alone				
No (ref)				
Yes	0·21	0·12, 0·30	0·21	0·12, 0·29
Education				
≤ 9 years (ref)				
≥ 10 years	−0·26	−0·33, –0·19	−0·25	−0·32, –0·19
Economic status				
Constrained (ref)				
Normal or good	−0·28	−0·34, –0·22	−0·18	−0·25, –0·12
Frequency of shopping				
1–2 times per week or less (ref)				
3–6 times per week or more	−0·16	−0·22, –0·10	−0·16	−0·22, –0·10

* The association of participants’ characteristics with DII scores was examined via regression analysis with energy intake as a covariate.

† Multiple regression analysis including all participants’ characteristics as explanatory variables was performed to examine the associations between the characteristics and DII scores. Energy intake was included as a covariate.

Table 3 shows the associations of the DII scores with disability and subjective health. In the entire population, the OR of disability was positively associated with the DII scores for overall disability (OR (95 % CI) = 1·23 (1·19, 1·28)) and disabilities in IADL (OR (95 % CI) = 1·10 (1·05, 1·15)), intellectual activities (OR (95 % CI) = 1·28 (1·23, 1·33)) and social participation (OR (95 % CI) = 1·17 (1·13, 1·22)). The OR for poor subjective health was also positively associated with DII scores (OR (95 % CI) = 1·09 (1·05, 1·14)). In the analysis stratified by municipality, DII scores were positively associated with the OR for overall disability and disabilities in intellectual activities and social participation (all six municipalities). Associations between DII scores and IADL disabilities were apparent only in Shimabara-shi and Hasami-cho, whereas associations with poor subjective health were seen only in Suzu-shi, Shimabara-shi, Matsuura-shi and Minamishimabara-shi.

**Table 3:** The associations of Dietary Inflammatory Index (DII) scores^(6)^ with overall disability and disabilities in each component of everyday competence assessed using the Tokyo Metropolitan Institute of Gerontology Index of Competence (TMIG-IC)^(21)^ and subjective health status (*n* 7930)^*^

		TMIG-IC									
		Overall		IADL		Intellectual activities		Social participation		Subjective health	
	*n*	OR	95 % CI	OR	95 % CI	OR	95 % CI	OR	95 % CI	OR	95 % CI
All municipalities	7930	1·23	1·19, 1·28	1·10	1·05, 1·15	1·28	1·23, 1·33	1·17	1·13, 1·22	1·09	1·05, 1·14
Ishikawa Prefecture											
Suzu-shi	1245	1·13	1·03, 1·23	1·10	0·98, 1·23	1·22	1·10, 1·34	1·11	1·02, 1·21	1·20	1·07, 1·34
Nagasaki Prefecture											
Unzen-shi	823	1·21	1·08, 1·35	1·04	0·90, 1·21	1·16	1·04, 1·30	1·14	1·02, 1·28	1·01	0·89, 1·15
Shimabara-shi	1025	1·30	1·17, 1·44	1·15	1·02, 1·32	1·29	1·16, 1·43	1·27	1·15, 1·40	1·14	1·01, 1·28
Hasami-cho	782	1·39	1·23, 1·58	1·44	1·20, 1·73	1·35	1·18, 1·56	1·28	1·12, 1·45	0·96	0·84, 1·11
Matsuura-shi	1253	1·28	1·17, 1·41	1·06	0·94, 1·21	1·37	1·25, 1·52	1·17	1·06, 1·28	1·12	1·01, 1·24
Minamishimabara-shi	2802	1·22	1·15, 1·30	1·07	0·99, 1·15	1·29	1·21, 1·37	1·17	1·10, 1·25	1·09	1·02, 1·17

IADL, instrumental activities of daily living.

* The associations of DII scores with overall disability, disabilities in each component of everyday competence (IADL, intellectual activities and social participation) and subjective health were determined via multiple logistic regression analyses using the data for the entire study population, followed by analyses stratified by municipality. Sex, age, BMI, educational and economic status and energy intake were included as covariates. The OR are for disability or poor subjective health. Overall disability was defined as an inability to perform at least one of the activities in the TMIG-IC questionnaire. Disability in a given component of everyday competence (IADL, intellectual activities and social participation) was defined as an inability to perform at least one of the activities in the corresponding component. The following question was used to assess subjective health: ‘In general, how do you feel about your health?’ Participants who answered (1) excellent or (2) good were considered to have good subjective health, while those who answered (3) poor or (4) very poor were considered to have poor subjective health.

## Discussion

The factors associated with inter-individual variations in DII scores were similar to those in our previous study^(14)^; in both studies, male sex, living alone, less education, lower economic status and lower shopping frequency were associated with higher DII scores. This implies that these factors and dietary inflammation may be commonly associated in the non-urban Japanese population.

Additionally, older age, higher BMI and residence in Matsuura-shi or Minamishimabara-shi were associated with higher DII scores. Previous Japanese studies reported lower DII scores in older participants^(11,12,19,20)^. Appetite loss associated with ageing can reduce the intake of certain pro-inflammatory nutrients, such as fat and protein, resulting in malnutrition^(27)^. Recent studies have suggested a bidirectional relationship between BMI and inflammation^(28)^. The higher DII scores in Matsuura-shi and Minamishimabara-shi may be explained by lower adherence to the typical Japanese diet, which is high in seafood and plant food and reduces mortality^(29)^. Lower intakes of the following anti-inflammatory nutrients best explained the higher DII scores in these municipalities: PUFA, *n*-6 fatty acids and vitamin D (all found in seafood) and dietary fibre and *β*-carotene (found in vegetables) (see online supplementary material, Supplemental Table 1). In Minamishimabara-shi, a higher fat intake (typical of a Western diet)^(30)^ also contributed to higher DII scores (see online supplementary material, Supplemental Table 1). Although moderate Westernisation of the traditional Japanese diet has improved the nutritional status of the postwar Japanese population^(29)^, certain features of a Western diet, such as low fibre intake and excessive fat intake, can promote inflammation^(30,31)^. The differences in nutrient intakes among municipalities highlight the importance of targeting populations that diverge from the typical Japanese diet, i.e. those following a Western diet, when planning public health interventions to reduce dietary inflammation in Japan.

DII scores were positively associated with both overall disability and components thereof, as reported in our previous study^(14)^. Other Japanese studies have linked DII scores mainly to disabilities in the basic functions required for everyday competence. One study used the disability certifications of the long-term care insurance system, which assesses impairments in physical and cognitive functions^(13)^. Others focused on sarcopenia, i.e. pathologically impaired physical function^(11)^, or frailty defined by the five components of the Cardiovascular Health Study Index^(12)^. In terms of everyday competence, two European studies reported associations between DII scores and IADL disabilities^(32,33)^, but few relevant studies have been performed in Asia (including Japan). Our study adds to previous knowledge, focusing on the inability to perform complex activities. This has received minimal attention, especially in Asian populations.

To the best of our knowledge, this is the first study to reveal an association between DII scores and subjective health. Poor subjective health may reflect prodromal disease status^(34)^ and has been associated with increased levels of C-reactive protein, an inflammatory biomarker^(35)^. Poor subjective health may predict future IADL disability^(15,16)^, and our study implies that dietary inflammation influences this risk factor.

Our analysis stratified by municipality yielded generally consistent results, strengthening our evidence. However, no associations of DII scores with IADL disability or subjective health were apparent in certain municipalities. Associations between DII scores and certain variables may thus be region-specific.

The strengths of our study include the relatively large sample size, the systematic sampling method employed and the younger age of the study population. We previously evaluated adults aged > 65 years^(14)^, whereas here we studied only those aged 65–74 years, who may be in earlier stages of disability development. The findings of this study should be interpreted in light of the following limitations. Due to the relatively moderate response rate (46 %), there is a potential for nonresponse bias in the study population, such as the healthy volunteer effect, where individuals who are healthier may be more likely to respond to the questionnaire. The potential issue of multiple testing is another limitation of this study. Given the multiple statistical analyses conducted across several health outcomes and subgroup analyses within individual municipalities, there is a potential risk of increased Type 1 errors (false positives), highlighting the need for careful interpretation of the findings.

Longitudinal studies are required to define causal relationships. In addition, the biological basis of the observed associations should be ascertained using measures of inflammatory biomarkers, including C-reactive protein.

### Conclusion

This study highlights the importance of reducing dietary inflammation in the early stage of disability development. We have identified the determinants of dietary inflammation in older Japanese adults living in non-urban municipalities, which may aid the development of appropriate interventions.

## Supporting information

Masuda et al. supplementary material Masuda et al. supplementary material

## Supplementary material

For supplementary material accompanying this paper visit https://doi.org/10.1017/S1368980025000424

## References

[1] Franceschi C & Campisi J (2014) Chronic inflammation (inflammaging) and its potential contribution to age-associated diseases. J Gerontol A Biol Sci Med Sci 69, S4–S9. 24833586 10.1093/gerona/glu057

[2] Furman D , Campisi J , Verdin E et al. (2019) Chronic inflammation in the etiology of disease across the life span. Nat Med 25, 1822–1832. 31806905 10.1038/s41591-019-0675-0 PMC7147972

[3] Noor S , Piscopo S & Gasmi A (2021) Nutrients interaction with the immune system. Arch Razi Inst 76, 1579–1588. 35546980 10.22092/ari.2021.356098.1775 PMC9083862

[4] Poles J , Karhu E , McGill M et al. (2021) The effects of twenty-four nutrients and phytonutrients on immune system function and inflammation: a narrative review. J Clin Transl Res 7, 333–376. 34239993 PMC8259612

[5] Ramos-Lopez O , Martinez-Urbistondo D , Vargas-Nuñez JA et al. (2022) The role of nutrition on meta-inflammation: insights and potential targets in communicable and chronic disease management. Curr Obes Rep 11, 305–335. 36258149 10.1007/s13679-022-00490-0 PMC9579631

[6] Shivappa N , Steck SE , Hurley TG et al. (2014) Designing and developing a literature-derived, population-based dietary inflammatory index. Public Heatlh Nutr 17, 1689–1696. 10.1017/S1368980013002115 PMC3925198 23941862

[7] Frith E , Shivappa N , Mann JR et al. (2018) Dietary inflammatory index and memory function: population-based national sample of elderly Americans. Br J Nutr 119, 552–558. 29361990 10.1017/S0007114517003804 PMC5839966

[8] Hayden KM , Beavers DP , Steck SE et al. (2017) The association between an inflammatory diet and global cognitive function and incident dementia in older women: the Women’s Health Initiative Memory Study. Alzheimers Dement 13, 1187–1196. 28531379 10.1016/j.jalz.2017.04.004 PMC5909961

[9] Kim D & Park Y (2018) Association between the Dietary Inflammatory Index and risk of frailty in older individuals with poor nutritional status. Nutrients 10, 1363. 30249038 10.3390/nu10101363 PMC6213380

[10] Shivappa N , Stubbs B , Hébert JR et al. (2018) The relationship between the dietary inflammatory index and incident frailty: a longitudinal cohort study. J Am Med Dir Assoc 19, 77–82. 28943182 10.1016/j.jamda.2017.08.006 PMC5756582

[11] Son BK , Akishita M , Yamanaka T et al. (2021) Association between inflammatory potential of the diet and sarcopenia/its components in community-dwelling older Japanese men. Arch Gerontol Geriatr 97, 104481. 34298260 10.1016/j.archger.2021.104481

[12] Son BK , Lyu W , Tanaka T et al. (2023) Impact of the anti-inflammatory diet on serum high-sensitivity C-Reactive protein and new-onset frailty in community-dwelling older adults: A 7-year follow-up of the Kashiwa cohort study. Geriatr Gerontol Int. Published online: 21 December 2023. doi: 10.1111/ggi.14781. PMC11503559 38126695

[13] Tomata Y , Shivappa N , Zhang S et al. (2018) Dietary Inflammatory Index and disability-free survival in community-dwelling older adults. Nutrients 10, 1896. 30513971 10.3390/nu10121896 PMC6315378

[14] Masuda M , Natsuhara K , Sueyoshi S et al. (2022) Association between the dietary inflammatory index and disability in Japanese older people. Public Health Nutr 25, 3137–3145. 35899875 10.1017/S1368980022001604 PMC9991663

[15] Fong JH & Kok ZC (2020) Does subjective health matter? Predicting overall and specific ADL disability incidence. Arch Gerontol Geriatr 90, 104169. 32645561 10.1016/j.archger.2020.104169

[16] Tas U , Verhagen AP , Bierma-Zeinstra SM et al. (2007) Prognostic factors of disability in older people: a systematic review. Br J Gen Pract 57, 319–323. 17394736 PMC2043327

[17] Tokudome S , Goto C , Imaeda N et al. (2004) Development of a data-based short food frequency questionnaire for assessing nutrient intake by middle-aged Japanese. Asian Pac J Cancer Prev 5, 40–43. 15075003

[18] Kotemori A , Sawada N , Iwasaki M et al. (2020) Validating the dietary inflammatory index using inflammatory biomarkers in a Japanese population: a cross- sectional study of the JPHC-FFQ validation study. Nutrition 69, 110569. 31574409 10.1016/j.nut.2019.110569

[19] Suzuki K , Shivappa N , Kawado M et al. (2020) Association between dietary inflammatory index and serum C-reactive protein concentrations in the Japan Collaborative Cohort Study. Nagoya J Med Sci 82, 237–249. 32581404 10.18999/nagjms.82.2.237 PMC7276400

[20] Yang Y , Hozawa A , Kogure M et al. (2020) Dietary Inflammatory Index positively associated with high-sensitivity C-reactive protein level in Japanese from NIPPON DATA2010. J Epidemiol 30, 98–107. 30745493 10.2188/jea.JE20180156 PMC6949183

[21] Koyano W , Shibata H , Nakazato K et al. (1987) Measurement of competence in the elderly living alone at home: development of an index of competence. Nihon Koshu Eisei Zasshi 34, 109–114.

[22] DeSalvo KB , Bloser N , Reynolds K et al. (2006) Mortality prediction with a single general self-rated health question. A meta-analysis. J Gen Intern Med 21, 267–275. 16336622 10.1111/j.1525-1497.2005.00291.x PMC1828094

[23] Okado J , Bin A , Tomoyama G et al. (2003) A follow-up study on the relationship between subjective health and mortality among the elderly people. Jpn Soc Health Educ Promot 11, 31–38.

[24] Crossley TF & Kennedy S (2002) The reliability of self-assessed health status. J Health Econ 21, 643–658. 12146595 10.1016/s0167-6296(02)00007-3

[25] Lundberg O & Manderbacka K (1996) Assessing reliability of a measure of self-rated health. Scand J Soc Med 24, 218–224. 8878376 10.1177/140349489602400314

[26] Zajacova A & Dowd JB (2011) Reliability of self-rated health in US adults. Am J Epidemiol 174, 977–983. 21890836 10.1093/aje/kwr204 PMC3218632

[27] Fielding RA , Landi F , Smoyer KE et al. (2023) Association of anorexia/appetite loss with malnutrition and mortality in older populations: a systematic literature review. J Cachexia Sarcopenia Muscle 14, 706–729. 36807868 10.1002/jcsm.13186 PMC10067499

[28] de Mello RN , de Gois BP , Kravchychyn ACP et al. (2023) Dietary inflammatory index and its relation to the pathophysiological aspects of obesity: a narrative review. Arch Endocrinol Metab 67, e000631. 37364142 10.20945/2359-3997000000631 PMC10661000

[29] Tsugane S (2021) Why has Japan become the world’s most long-lived country: insights from a food and nutrition perspective. Eur J Clin Nutr 75, 921–928. 32661353 10.1038/s41430-020-0677-5 PMC8189904

[30] Malesza IJ , Malesza M , Walkowiak J et al. (2021) High-fat, western-style diet, systemic inflammation, and gut microbiota: a narrative review. Cells 10, 3164. 34831387 10.3390/cells10113164 PMC8619527

[31] Patry RT & Nagler CR (2021) Fiber-poor western diets fuel inflammation. Nat Immunol 22, 266–268. 33574618 10.1038/s41590-021-00880-x

[32] Laclaustra M , Rodriguez-Artalejo F , Guallar-Castillon P et al. (2020) The inflammatory potential of diet is related to incident frailty and slow walking in older adults. Clin Nutr 39, 185–191. 30737049 10.1016/j.clnu.2019.01.013

[33] Assmann KE , Adjibade M , Shivappa N et al. (2018) The inflammatory potential of the diet at midlife is associated with later healthy aging in French adults. J Nutr 148, 437–444. 29546305 10.1093/jn/nxx061 PMC6251567

[34] Idler EL & Benyamini Y (1997) Self-rated health and mortality: a review of twenty-seven community studies. J Health Soc Behav 38, 21–37. 9097506

[35] Leshem-Rubinow E , Shenhar-Tsarfaty S , Milwidsky A et al. (2015) Self-rated health is associated with elevated C-reactive protein even among apparently healthy individuals. Isr Med Assoc J 17, 213–218. 26040045

