# Supplementary material for: Associations of Dietary Inflammatory Index Scores with the disability status and subjective health of older adults living in non-urban municipalities in Nagasaki and Ishikawa Prefectures, Japan
Source: Public Health Nutr. 2025 Mar 31;28(1):e71. doi: 10.1017/S1368980025000424 (PMC12086721; doi:10.1017/S1368980025000424)
Supplement: Masuda et al. supplementary material [file S1368980025000424sup001.docx]

| **Supplementary Table 1. Anti- and pro-inflammatory food parameters used to calculate Dietary Inflammatory Index (DII) scores (n = 7930).*** | | | | | | | | | | | | | | | |
| --- | --- | --- | --- | --- | --- | --- | --- | --- | --- | --- | --- | --- | --- | --- | --- |
| Food Parameters | All municipalities  (n = 7930) | | SZ  (n = 1245) | | UZ  (n = 823) | | SB  (n = 1025) | | HM  (n = 782) | | MU  (n = 1253) | | MS  (n = 2802) | | *p*^§^ |
|  | Intake^†^ | DII^‡^ | Intake^†^ | DII^‡^ | Intake^†^ | DII^‡^ | Intake^†^ | DII^‡^ | Intake^†^ | DII^‡^ | Intake^†^ | DII^‡^ | Intake^†^ | DII^‡^ |  |
| Anti-inflammatory food parameters | | | | | | | | | | | | | | | |
| Dietary fiber (g) | 11.47 | 0.51 | 12.19 | 0.48 | 11.24 | **0.52^A^** | 11.61 | 0.50 | 11.37 | **0.52^A^** | 10.93 | **0.53^A^** | 11.43 | **0.51^A^** | **<0.01** |
| Alcohol (g) | 2.99 | 0.24 | 3.81 | 0.22 | 2.71 | **0.24^A^** | 2.65 | **0.24^A^** | 3.31 | **0.23^A^** | 3.16 | **0.23^A^** | 2.66 | **0.24^A^** | **<0.01** |
| β-carotene (μg) | 3091.55 | 0.17 | 3167.33 | 0.15 | 3093.08 | 0.17 | 3204.24 | 0.14 | 3197.77 | 0.15 | 2980.88 | **0.19^A^** | 3036.05 | **0.18^A^** | **<0.01** |
| Vitamin C (mg) | 97.88 | 0.14 | 99.52 | 0.13 | 98.60 | 0.13 | 99.75 | 0.13 | 98.84 | 0.13 | 96.08 | 0.15 | 96.78 | 0.14 | 0.10 |
| Vitamin E (mg) | 8.23 | 0.10 | 8.02 | 0.13 | 8.34 | 0.10 | 8.44 | **0.08^B^** | 8.30 | 0.10 | 8.02 | 0.12 | 8.30 | **0.09^B^** | **<0.01** |
| Thiamin (mg) | 0.69 | 0.09 | 0.69 | 0.08 | 0.69 | 0.08 | 0.69 | 0.09 | 0.69 | 0.09 | 0.68 | 0.09 | 0.69 | 0.09 | 0.17 |
| Riboflavin (mg) | 1.17 | 0.03 | 1.08 | 0.04 | 1.17 | **0.03^B^** | 1.19 | **0.03^B^** | 1.23 | **0.03^B^** | 1.16 | **0.03^B^** | 1.18 | **0.03^B^** | **<0.01** |
| Vitamin A (RE) | 1009.26 | 0.03 | 866.73 | 0.08 | 1058.87 | **0.01^B^** | 1060.83 | **0.00^B^** | 1041.70 | **0.01^B^** | 987.90 | **0.04^B^** | 1039.66 | **0.01^B^** | **<0.01** |
| PUFA (g) | 13.81 | 0.02 | 14.58 | -0.01 | 13.61 | **0.04^A^** | 13.79 | **0.02^A^** | 13.88 | **0.02^A^** | 13.20 | **0.05^A^** | 13.79 | **0.02^A^** | **<0.01** |
| MUFA (g) | 17.05 | 0.01 | 16.58 | 0.01 | 17.09 | 0.01 | 17.35 | 0.01 | 17.34 | 0.01 | 16.68 | 0.01 | 17.21 | 0.01 | **0.02** |
| *n-*6 fatty acids(g) | 11.51 | -0.01 | 12.15 | -0.02 | 11.40 | **-0.01^A^** | 11.59 | **-0.01^A^** | 11.55 | **-0.01^A^** | 10.98 | **0.00^A^** | 11.46 | **-0.01^A^** | **<0.01** |
| Folic acid (μg) | 335.02 | -0.06 | 310.03 | -0.02 | 345.44 | **-0.06^B^** | 341.93 | **-0.06^B^** | 353.99 | **-0.08^B^** | 335.05 | **-0.06^B^** | 335.23 | -0.05 | **<0.01** |
| Vitamin D (μg) | 7.62 | -0.06 | 8.25 | -0.09 | 7.48 | **-0.03^A^** | 6.91 | **-0.01^A^** | 7.67 | -0.07 | 7.66 | **-0.06^A^** | 7.61 | **-0.06^A^** | **<0.01** |
| *n*-3 fatty acids (g) | 3.06 | -0.37 | 3.17 | -0.37 | 3.04 | -0.37 | 2.96 | -0.37 | 3.08 | -0.37 | 3.04 | -0.37 | 3.06 | -0.37 | **0.04** |
| Pro-inflammatory food parameters | | | | | | | | | | | | | | | |
| Protein (g) | 58.26 | -0.02 | 59.74 | -0.01 | 57.64 | -0.02 | 57.03 | **-0.02^B^** | 58.44 | -0.02 | 57.27 | -0.02 | 58.63 | -0.01 | **0.03** |
| Cholesterol (mg) | 269.07 | -0.02 | 278.14 | -0.01 | 263.36 | **-0.02^B^** | 270.05 | -0.01 | 272.96 | -0.01 | 265.69 | **-0.02^B^** | 266.77 | **-0.02^B^** | **<0.01** |
| Iron (mg) | 7.59 | -0.03 | 7.94 | -0.02 | 7.48 | **-0.03^B^** | 7.45 | **-0.03^B^** | 8.01 | -0.02 | 7.49 | **-0.03^B^** | 7.44 | **-0.03^B^** | **<0.01** |
| Carbohydrate (g) | 247.29 | -0.03 | 247.60 | -0.03 | 247.59 | -0.03 | 239.93 | -0.03 | 245.95 | -0.03 | 249.29 | -0.02 | 249.23 | -0.02 | **<0.01** |
| Total fat (g) | 47.10 | -0.21 | 45.86 | -0.22 | 46.76 | -0.21 | 48.31 | **-0.20^A^** | 47.68 | **-0.21^A^** | 45.67 | -0.22 | 47.79 | **-0.20^A^** | **<0.01** |
| Saturated fat (g) | 12.04 | -0.35 | 11.91 | -0.35 | 11.99 | -0.35 | 12.14 | -0.35 | 12.19 | -0.35 | 11.70 | -0.35 | 12.19 | -0.35 | 0.19 |

SZ, Suzu-shi, Ishikawa Prefecture

UZ, Unzen-shi, Nagasaki Prefecture

SB, Shimabara-shi, Nagasaki Prefecture

HM, Hasami-cho, Nagasaki Prefecture

MU, Matsuura-shi, Nagasaki Prefecture

MS, Minamishimabara-shi, Nagasaki Prefecture

*Adjustment for energy intake was not possible.

^†^Mean intakes of various foods parameters as estimated by answers to the food frequency questionnaire.

^‡^Mean food parameter-specific DII scores.

^§^Differences in food parameter-specific DII scores among the six municipalities were evaluated by analysis of variance (ANOVA). The level of statistical significance was set at *P* < 0.05.

^A, B^ The food parameter-specific DII scores of the five municipalities in Nagasaki Prefecture were compared to those of Suzu-shi (Ishikawa Prefecture) using the Dunnett test; the data for Suzu-shi served as the reference. Significantly higher values (compared to those of Suzu-shi) are marked ‘A’, and significantly lower values are marked ‘B’. The level of statistical significance was *P* < 0.05.
